# Food allergy, a summary of recent cases in the criminal and civil courts of the UK

**DOI:** 10.1186/2045-7022-1-S1-O1

**Published:** 2011-08-12

**Authors:** Hazel M Gowland, Michael J Walker

**Affiliations:** 1 Allergy Action, St Albans, UK; 2 LGC, Teddington, UK

## 

Food allergy, a significant public health concern in the developed world has developed a forensic context where allergy related personal injury, fatality or criminal non-compliance on the part of a food supplier are issues that have recently come before the UK courts. EU General Food Law (178/2002/EC) and Directive 2000/13/EC (to be consolidated into the proposed Food Information Regulation) address allergen avoidance risks relating to composition, labelling and food safety. The European Framework Directive on Safety and Health at Work (Directive 89/391 EEC and daughter legislation) may also be deployed. Compensation in civil law for loss or damage caused by an allergic reaction to a food supplied, not as requested, or with misleading or incorrect information has also been sought. The authors describe recent such cases dealing with prepacked retail and non prepacked catering situations where contraventions of food law could have resulted in fatalities, and health and safety and civil litigation following deaths from food allergy. The cases came before the civil (including appeal) courts, magistrates and coroner’s courts. In the absence of a current cure, health protection depends on accurate diagnosis, informed food allergen avoidance and the effective management of symptoms. These strategies depend in turn on the multilayered approaches adopted by clinicians, scientists, food suppliers, allergic consumers (and those who care for them), and regulators. Effective food allergen avoidance depends on the diagnosed consumer knowing what to avoid, being able to access correct ingredients information and to rely on the absence of allergen cross-contamination. Access to ingredients information may be via a label, menu or other printed means, or by enquiring of a staff member who may not always appreciate the implications of such a request or be competent in managing food allergy risks. Lapses in any of these areas may have forensic consequences beyond even their initial (sometimes severe) personal impact.

